# Cognition-enhancing drugs induce intrusive traumatic memories: a two-case report

**DOI:** 10.1002/ccr3.82

**Published:** 2014-05-23

**Authors:** Nadine Zahlan, Anthony Levitt

**Affiliations:** 1 Department of Psychiatry, Sunnybrook Health Sciences CentreToronto, Ontario, Canada; 2 Institute of Health Policy, Management and Evaluation, University of TorontoToronto, Ontario, Canada; 3 Department of Psychiatry, University of TorontoToronto, Ontario, Canada

**Keywords:** Adverse drug reaction, adverse events, cognition-enhancing drugs, patient safety, psychiatry

## Abstract

**Key Clinical Message:**

Physicians managing patients with the use of drugs with cognition-enhancing properties should be aware of the possibility of concurrent emergent, intrusive traumatic memories in individuals without existing cognitive impairment. Dose response should be closely monitored, as the adverse event may follow titration past an individual threshold.

## To the editor

We report two cases of drug-induced traumatic and intrusive memories in female patients with a history of chronic pain receiving treatment for a mood disorder. In both cases, these memories emerged after a dosage increase, and disappeared immediately with drug cessation. The first patient received donepezil, an acetylcholinesterase inhibitor, which has previously been reported to induce posttraumatic memories. The second patient received lurasidone, a novel atypical antipsychotic with cognition-enhancing properties but for which no reports of induced traumatic memories exist.

## Case 1

Mrs. X was a 52-year-old female with a 12-year treatment history for chronic discogenic pain at L3 and L4; she had concurrent bipolar disorder. Mrs. X had begun to complain of worsening cognition, including word-finding and general computational difficulties. Mrs. X was prescribed donepezil 2.5 mg in addition to her mood stabilizing medications for the cognitive difficulties with no noticeable effects. At 5 mg, she reported making fewer mistakes when answering phone calls at work and an increased available vocabulary within a week. However, she also reported debilitating flooding of intrusive and traumatic memories about a motor vehicle accident 13 years previously. Mrs. X had never reported intrusive memories or significant distress at any time in the previous 13 years.

Within 48 h of discontinuing donepezil, Mrs. X reported a complete resolution of intrusive memories and distress; within a week her cognitive impairment returned to pretreatment levels.

## Case 2

Mrs. Y was a 52-year-old retired nurse with a 15-year treatment history for chronic neuropathic spinal pain and concurrent major depression with significant anhedonia. Over 15 years, Mrs. X had tried multiple psychotropic medications and combinations that had been unsuccessful in significantly reducing her depression or chronic pain. Aripiprazole 10 mg was added to her treatment plan, after which, Mrs. Y reported less irritability but also cognitive dullness and a lack of creativity. Lurasidone was subsequently substituted for aripiprazole with the intention of resolving its apparent cognitive side effects.

After commencing lurasidone 10 mg, Mrs. Y reported a 50% reduction in pain, improved mood and enhanced cognition; she was “reading voraciously” for the first time in years. Two weeks later, lurasidone was increased to 20 mg and Mrs. Y reported further reduction in her pain, improvement in mood, and cognition. However, she also reported new onset of distress around her sister-in-law's recent breast cancer diagnosis. Lurasidone was increased to 25 mg and within 2 days, Mrs. Y experienced flooding of debilitating and intrusive traumatic memories of an ICU patient's death 20 years previously and the remote death of her mother-in-law. Mrs. Y had no prior history of intrusive or traumatic memories regarding these events.

Despite significant debilitation and distress, Mrs. X was reluctant to discontinue treatment with lurasidone. At the advice of her physician, it was nonetheless discontinued. Within 24 h of discontinuing lurasidone, Mrs. Y's distress and traumatic memories completely resolved; over several days, her pain and low mood returned to its pretreatment level.

## Discussion

Although donepezil and lurasidone belong to distinct pharmacological classes, both have been implicated in enhanced cognition when baseline cognitive impairment exists. Lurasidone is new to the market and little is known about its mechanisms beyond the fact that it has the highest affinity for 5-HT7 in its class. Similarly, little is known about the mechanisms of 5-HT7, although it has been preliminarily implicated in enhanced cognition via its ability to reverse pharmacologically induced cognitive impairment in rat and mice models [1,2]. Acetylcholinesterase inhibitors, like donepezil, have comparably reduced scopoloamine-induced amnesia in rats [3].

The two cases presented in this report show striking similarities in the timing of onset and offset of effects on memory and cognition, which to our knowledge, have never been reported in individuals without evidence of a neurodegenerative condition. There may be several explanations for the triggering of intrusive memories without existing impairment, as in the current cases. It is possible that donepezil and lurasidone activated or further enhanced memory function; and/or, they may have decreased the ability to filter or inhibit unwanted memories; and/or, in Mrs. Y's case specifically, lurasidone may have resulted in increased obsessionality, as has been noted with other atypical antipsychotics [4]. However, unlike previous reports of atypical antipsychotic-induced obsessive symptoms, Mrs Y had no preexisting obsessionality.

In both the cases, the patients experienced such profound distress and preoccupation with the intrusive memories such that, rather than downward titration, the treating physician immediately discontinued drug administration out of concern for patient safety. However, as the traumatic memories succeeded the moderately enhanced cognition (i.e., in Mrs. X's improved work performance and Mrs. Y's increased reading ability) and emerged immediately after reaching an upwardly titrated dosage threshold, future investigations in a clinically controlled environment should aim to determine whether downward titration successfully resolves this occurrence.

Taken together, these novel cases highlight the nonspecific cognition-enhancing effects of these agents. Further investigations into this possible effect and its resolution are warranted as these cases also demonstrate the potential for cognition-enhancing agents to relieve treatment-resistant chronic pain, improve mood, and cognitive performance. In Mrs. Y's case, the treating physician importantly notes her hesitance to discontinue treatment, despite extreme distress, because of lurasidone's success in reducing her previously treatment-resistant chronic pain. The induction of intrusive, distressing memories by cognition-enhancing agents that are new to the market have significant clinical relevance and treatment implications; this report, therefore, adds to the modest existing knowledge base and highlights the need for physicians to be acutely aware of the potential for such symptoms.

## References

[1] Roberts AJ, Hedlund PB (2012). The 5-HT(7) receptor in learning and memory. Hippocampus.

[2] Meneses A (2004). Effects of the 5-HT_7_ receptor antagonists SB-269970 and DR 4004 in autoshaping Pavlovian/instrumental learning task. Behav. Brain Res.

[3] Wong KK (2010). Cryptotanshinone, an acetylcholinesterase inhibitor from Salvia miltiorrhiza, ameliorates amnesia in Morris water maze task. Planta Med.

[4] Khullar A, Chue P, Tibbo P (2001). Quetiapine and obsessive-compulsive symptoms (OCS): case report and review of atypical antipsychotic-induced OCS. J. Psychiatry Neurosci.

